# A gold cluster fused manganese dioxide nanocube loaded with dihydroartemisinin for effective cancer treatment *via* amplified oxidative stress†

**DOI:** 10.1039/d4ra03164d

**Published:** 2024-09-02

**Authors:** Alan Tianyi Wang, Xin Wen, Shangyi Duan, Jiangwei Tian, Liang Liu, Wangning Zhang

**Affiliations:** a State Key Laboratory of Natural Medicines, Jiangsu Key Laboratory of TCM Evaluation and Translational Research, School of Traditional Chinese Pharmacy, China Pharmaceutical University 639 Longmian Avenue Nanjing 211198 China jwtian@cpu.edu.cn 18435148209@163.com; b Shanghai Xianguang Biotechnology Co., Ltd 318 Jungong Road Shanghai 200090 China stephen@xgbiotech.com

## Abstract

Chemodynamic therapy, leveraging metabolic processes for reactive oxygen species (ROS) generation, shows promise in cancer eradication. However, its efficacy is hampered by hypoxic conditions, substrate scarcity, and abundant ROS scavengers. In this study, we have devised a cubic manganese oxide nanozyme (BSA–AuNC–MnO_2_@DHA) to tackle these obstacles. This nanozyme integrates MnO_2_ with bovine serum albumin (BSA)-coated gold nanoclusters (AuNC), forming BSA–AuNC–MnO_2_, and further incorporates dihydroartemisinin (DHA) to confer both bioimaging and anticancer capabilities. The BSA–AuNC–MnO_2_ nanoparticles exhibit a uniform cubic morphology, with an average hydrated particle diameter of 76.4 ± 7.1 nm and a zeta potential of −32.6 mV, indicative of their excellent dispersion and stability. The encapsulation efficiency of DHA within the BSA–AuNC–MnO_2_@DHA system achieved a remarkable value of 72.45%, attesting to its substantial drug-loading capacity. MnO_2_ serves a dual function within the nanozyme: it augments oxidative stress while concurrently inhibiting antioxidant defenses. It depletes the antioxidant glutathione (GSH) to release Mn^2+^, which in turn catalyzes ROS production from intracellular substrates and DHA. The remarkable anticancer efficacy of this tailored approach is evidenced by the potent inhibition of tumor growth observed after a single-dose administration, which underscores the amplification of oxidative stress. Additionally, BSA–AuNC–MnO_2_@DHA exhibits negligible toxicity to major organs, highlighting its exceptional biocompatibility and safety profile.

## Abstract 2

A multifunctional nanomedicine has been developed to treat cancer cells by amplifying oxidative stress and integrating bioimaging for therapeutic monitoring.

## Graphical abstract

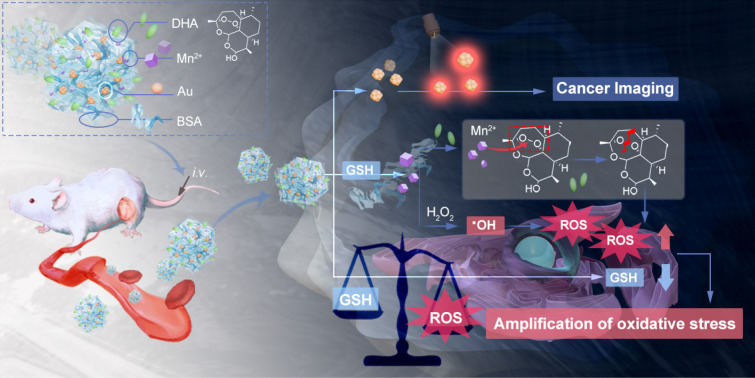

## Introduction

Cancer stands as a paramount global threat to human health.^1^ Conventional cancer therapies, including chemotherapy, radiation therapy, and surgery, inevitably cause side effects, damage healthy organs/tissues, and compromise the patients' health. As an alternative strategy, cancer oxidative therapy has emerged, leveraging intrinsic oxidative stress to elicit targeted cell death.^2,3^ This approach involves the generation of excessive reactive oxygen species (ROS), disrupting intracellular redox homeostasis and inflicting harm on crucial cellular constituents, such as protein, DNA, and lipids.^4^ The cumulative damage subsequently triggers programmed cancer cell death mechanisms, including apoptosis and necrosis.^5,6^ Among oxidative therapies, chemodynamic therapy (CDT) stands out, harnessing Fenton or Fenton-like reactions to convert endogenously abundant but less-reactive hydrogen peroxide (H_2_O_2_) into highly potent hydroxyl radicals (˙OH) in the presence of metal catalysts.^7–10^ The utilization of naturally occurring hydrogen peroxide imparts CDT with exceptional biocompatibility. Moreover, CDT is selective for cancer over normal tissues due to the production of ˙OH, which highly relies on the tumor's endogenous microenvironment, low pH, and elevated H_2_O_2_ levels.^11^ Consequently, CDT's blend of biocompatibility and tumor specificity positions it as a promising avenue for cancer treatment.

In the realm of materials utilized for CDT, nanozymes^12–18^ especially MnO_2_ nanozymes, distinguish themselves through their unparalleled stability, exceptional biocompatibility, facile modification capabilities, and cost-effectiveness.^19–21^ The versatility in MnO_2_ preparation as nanoparticles, nanowires, nanosheets, and nanoflowers expands the application in biosensing, bioimaging, and drug delivery.^22–25^ As a self-amplifying nanoreactor, the most attractive point of MnO_2_ for CDT is the depletion of glutathione (GSH).^26^ As an endogenous antioxidant, GSH scavenges the highly reactive ˙OH, hampering the efficiency of CDT. Furthermore, MnO_2_ nanosystems have garnered attention for their potential to accomplish multiple therapeutic goals concurrently,^27^ including targeted oxygen generation in tumor microenvironments (TME), GSH depletion, and biodegradability, thereby offering a promising multifaceted approach.^28–30^ Numerous ROS-generating nanomedicines, incorporating Fe^2+^ or Fe^3+^, as well as non-ferrous components exhibiting versatile oxidation states, notably Mn^2+^/Mn^4+^ and Cr^3+^/Cr^6+^, have garnered significant attention as potent Fenton reaction catalysts. These nanodrugs efficiently degrade H_2_O_2_ into highly reactive ˙OH, thereby initiating cellular demise. A prominent instance lies in the redox interplay between GSH and MnO_2_, which has emerged as a promising avenue for innovative cancer treatment strategies, fostering both research and exploration in this domain.^31^ However, enhancing the efficacy of MnO_2_ is imperative for the establishment of an effective cancer-killing modality. Consequently, efficacious MnO_2_-based therapeutic strategies are routinely integrated with additional therapeutic agents to amplify their overall effectiveness.

**Scheme 1:**
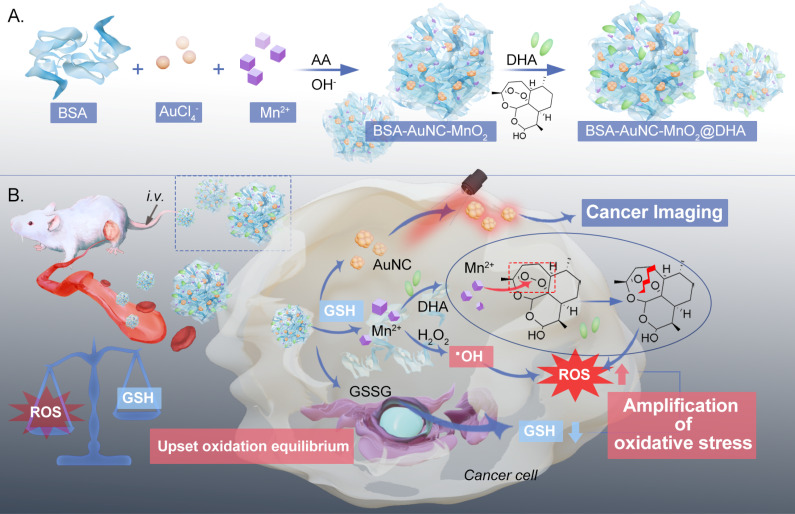
Schematic representation for (A) synthesis of BSA–AuNC–MnO_2_@DHA and (B) its application in cancer imaging and effective treatment *via* amplification of oxidative stress by elevating the level of ROS and reducing the level of GSH. (BSA: bovine serum albumin; AA: ascorbic acid; DHA: dihydroartemisinin; GSH: glutathione; GSSG: glutathione oxidized; AuNC: gold nanocluster).

In this study, we devised a cubic gold nanocluster-fused MnO_2_-based nanozyme, denoted as BSA–AuNC–MnO_2_@DHA, which encapsulates dihydroartemisinin (DHA) for the enhancement of oxidative therapy (Scheme 1). This nanozyme integrates bovine serum albumin (BSA)-stabilized gold nanoclusters (AuNCs) for enhanced biocompatibility and bioimaging capabilities with MnO_2_, functioning as the nanozymatic component. The incorporation of DHA within the nanoconstruct serves to elicit an overproduction of ROS. Specifically tailored for cancer cells rich in GSH, this cubic nanozyme exerts a dual-pronged effect: it concurrently catalyzes ROS generation and depletes GSH through the action of MnO_2_ nanozymes. Consequently, GSH depletion triggers the release of Mn^2+^, which react with DHA, rupturing its endoperoxide bridge to generate toxic alkoxyl radicals, thereby fostering cancer cell eradication.^32,33^ Notably, this targeted therapeutic approach minimizes adverse effects on healthy cells while inflicting substantial damage on cancerous ones, effectively arresting tumor progression. Consequently, BSA–AuNC–MnO_2_@DHA emerges as a promising “all-in-one” nanomedicine platform for selective cancer therapy.

## Results and discussion

**Fig. 1:**
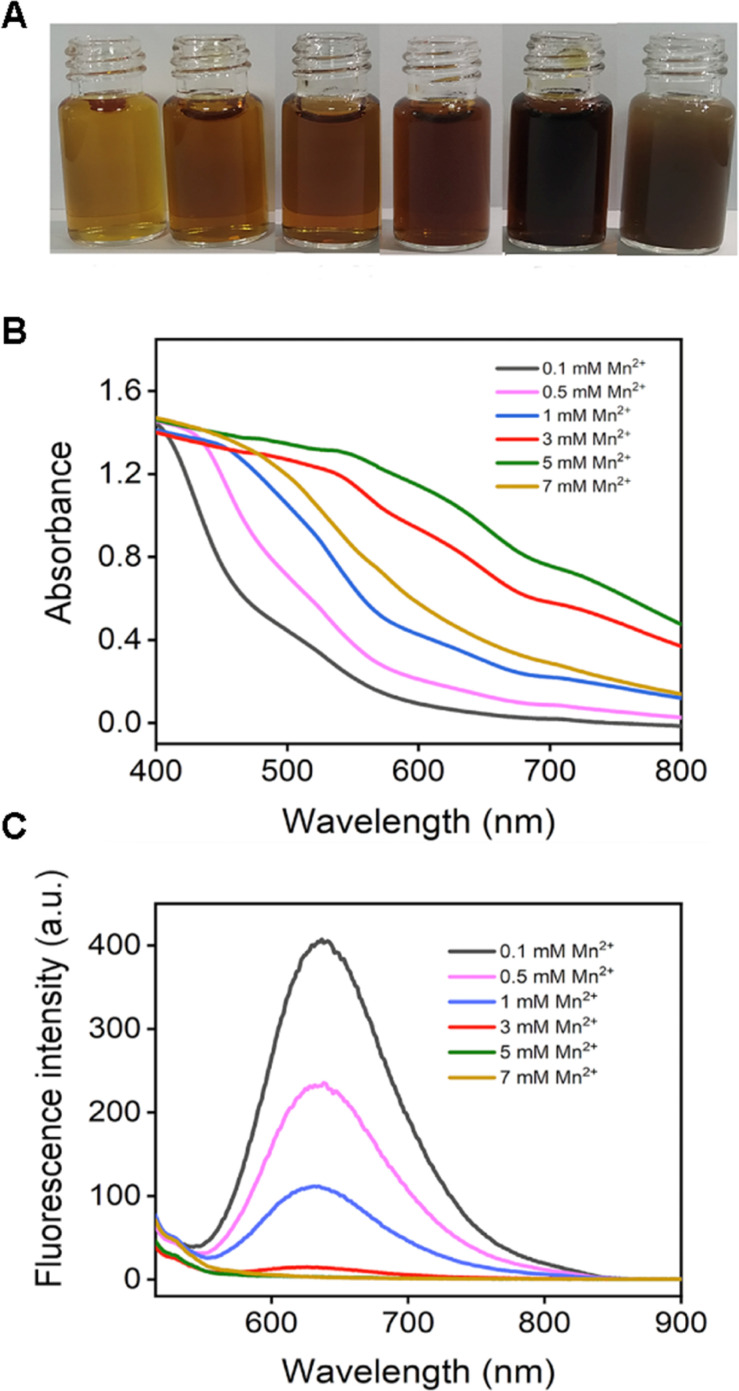
Screening Mn^2+^ on BSA–AuNC–MnO_2_ synthesis. (A) Photos of BSA–AuNC–MnO_2_ synthesized at different concentrations of Mn^2+^. (B) UV-Vis-NIR spectra of different formulations of BSA–AuNC–MnO_2_. (C) Fluorescence spectra of different formulations of BSA–AuNC–MnO_2_ (excited at 495 nm).

The cubic structure was provided by BSA–AuNC, presumably due to the partial denaturation of BSA during the synthesis.^34^ The optimization of BSA–AuNC, achieved by adjusting the BSA to HAuCl_4_ ratio, pH, temperature, and reaction time, significantly enhanced its stability and functionality (Fig. S1–S7,† synthesis was mentioned in the ESI Method section†). Specifically, the optimal formulation, consisting of 10 mg per mL BSA, 5 mM HAuCl_4_, and 20 μM ascorbic acid reduced at 100 °C under a pH of 12.10 for two hours, yielded cubic BSA–AuNCs with enhanced near-infrared (NIR) fluorescence properties. BSA–AuNC–MnO_2_ was then synthesized by fusing Mn^2+^ into the cubic BSA-coated AuNC using ascorbic acid as the reducing agent. The final range of Mn^2+^ concentration varied from 0 mM to 7 mM. Visual observation of the optical image (Fig. 1A) revealed slight precipitation of BSA–AuNC–MnO_2_ synthesized using 7 mM and 5 mM concentrations. The precipitation corresponded to the outliers in the absorbance spectra, while the spectra of other BSA–AuNC–MnO_2_ were consistent with increasing concentrations (Fig. 1B). The incorporation of Mn was associated with the quench of NIR fluorescence (Fig. 1C) due to the broad UV absorption. A minimal fluorescence in 3 mM Mn^2+^ indicated 3 mM Mn^2+^ was the optimal condition for BSA–AuNC–MnO_2_ formulation. These findings highlight the importance of precise control over synthesis conditions and Mn^2+^ dosing in achieving the desired properties of the nanocomplexes. Collectively, these findings underscore the paramount importance of rigorous optimization procedures and meticulous control over the synthesis conditions in the production of highly stable and multifunctional nanomaterials. The successful integration of Mn^2+^ into BSA–AuNCs, yielding BSA–AuNC–MnO_2_ nanocomplexes that exhibit tunable fluorescence properties alongside potential catalytic activities, presents novel opportunities for exploring the utilization of these materials across diverse fields, including biosensing, imaging technologies, and drug delivery systems.

**Fig. 2:**
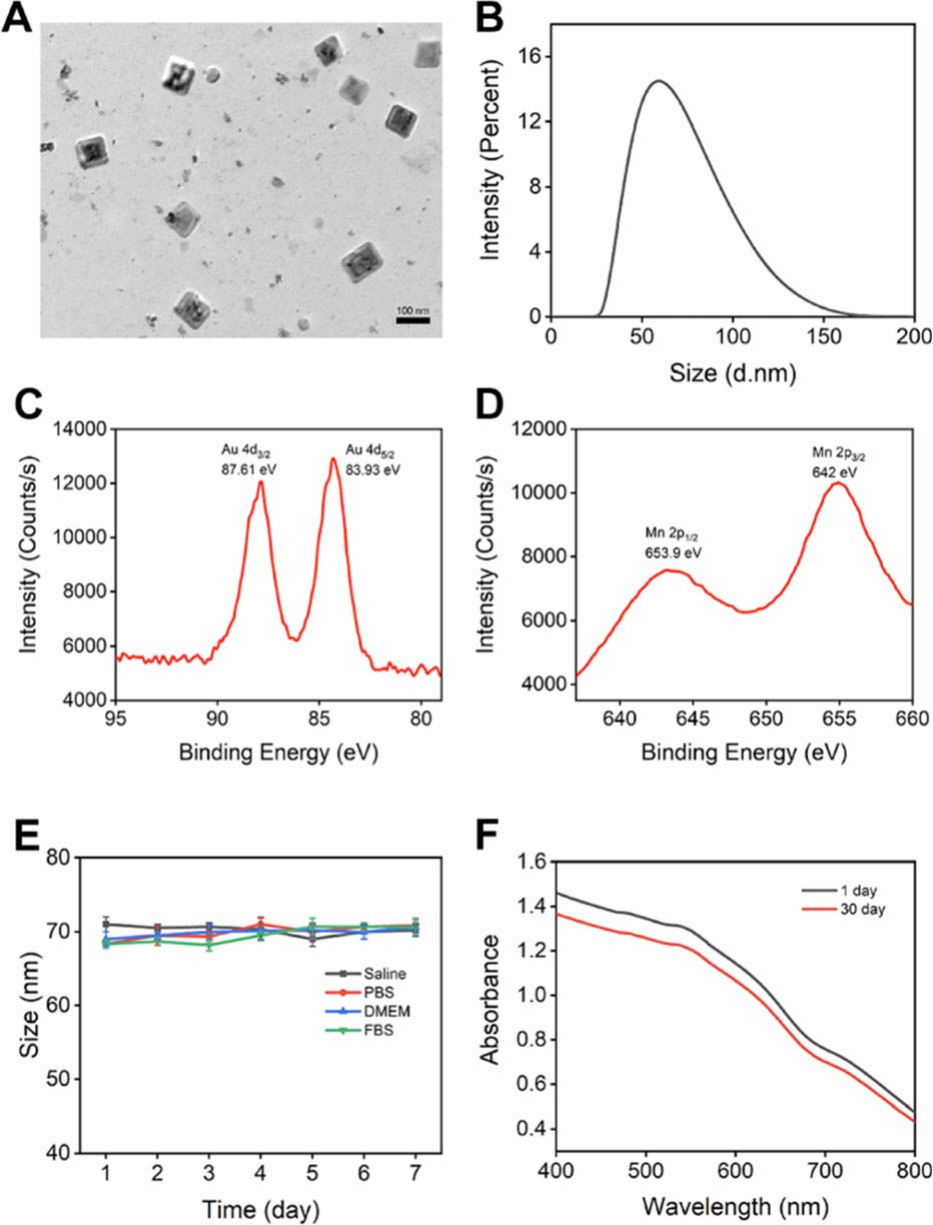
Characterization of BSA–AuNC–MnO_2_. (A) TEM image of BSA–AuNC–MnO_2_ (scale bar = 100 nm). (B) Dynamic light scatting (DLS) of BSA–AuNC–MnO_2_. XPS spectra of 4d orbital from Au (C) and 2p orbital from Mn (D). (E) Sizes of BSA–AuNC–MnO_2_ in different solutions over 7 days. (F) UV-Vis-NIR spectra of BSA–AuNC–MnO_2_ on day 1 and day 30.

The synthesized BSA–AuNC–MnO_2_ nanoparticles exhibited a uniform size of 76.4 ± 7.1 nm and 62 nm, as corroborated by transmission electron microscopy (TEM) and dynamic light scattering (DLS) analyses (Fig. 2A and B). This size falls within the optimal range conducive to enhanced cellular uptake and biodistribution. Notably, the zeta potential of the nanoparticles was measured to be −32.6 mV (Fig. S8†), further underscoring their stability and potential for effective interaction with biological systems. The TEM images revealed a uniform distribution of cubic-shaped nanoparticles, which is particularly indicative of their potential to facilitate enhanced cellular penetration. Moreover, the successful synthesis of BSA–AuNC–MnO_2_ was unequivocally demonstrated through the presence of distinct peaks in the X-ray photoelectron spectroscopy (XPS) spectra. Additionally, we adjusted the synthesis method based on the literature to achieve higher purity,^35^ resulting in a particle size of 72.1 ± 2.9 nm (Fig. S9†).

As shown in Fig. 2C and D, the peaks observed at 87.61 eV and 83.93 eV correspond to the 4p orbital of gold (Au), whereas the peaks at 653.9 eV and 642 eV are attributed to the 2p orbital of manganese. These findings provide compelling evidence for the successful doping of Mn into the AuNCs, as corroborated by the binding energies derived from XPS analysis. Overall, this synthesis strategy has produced well-defined BSA–AuNC–MnO_2_ nanoparticles that possess optimal physicochemical properties, including size, stability, and composition. These attributes position them as promising candidates for further exploration into their potential biomedical applications, where their enhanced cellular uptake, biodistribution, and potential for interaction with biological systems could be harnessed for therapeutic, diagnostic, or imaging purposes.

The quantitative evaluation of hydroxyl radical (˙OH) generation and subsequent GSH depletion, facilitated by BSA–AuNC–MnO_2_, was conducted by meticulously monitoring the degradation kinetics of methylene blue (MB) amidst varying concentrations of GSH. Our findings revealed a marked decline in MB concentration upon exposure to BSA–AuNC–MnO_2_, accompanied by a corresponding rise in GSH levels, emphasizing the system's potent capacity to produce ˙OH for CDT (in Fig. S10†). Notably, the introduction of 10 mM GSH partially alleviated the observed decrease in absorbance, attributable to GSH's inherent antioxidant properties, thereby providing valuable insights into its regulatory role in mitigating the oxidative stress induced by the system.

The pivotal significance of stability in the biological utilization of BSA–AuNC–MnO_2_ nanomaterials is underscored by rigorous assessment in diverse aqueous solutions and cell culture environments (Fig. 2E). Our findings reveal an extraordinary stability of the nanocubes, as evidenced by the absence of notable alterations in particle dimensions over a seven-day period, thereby emphasizing their robust endurance within biological milieus. To further corroborate this stability, an extensive temporal evaluation was conducted, monitoring the UV-Vis-NIR spectroscopic signatures (Fig. 2F). The minimal spectral variations observed between the initial day and the 30th day underscore the remarkable preservation of the nanocubes' optical characteristics over an extended timeframe. This paramount stability was crucial in ensuring the sustained bioavailability of the nanozyme, enabling it to maintain its functional integrity and dimensional consistency over time.

**Fig. 3:**
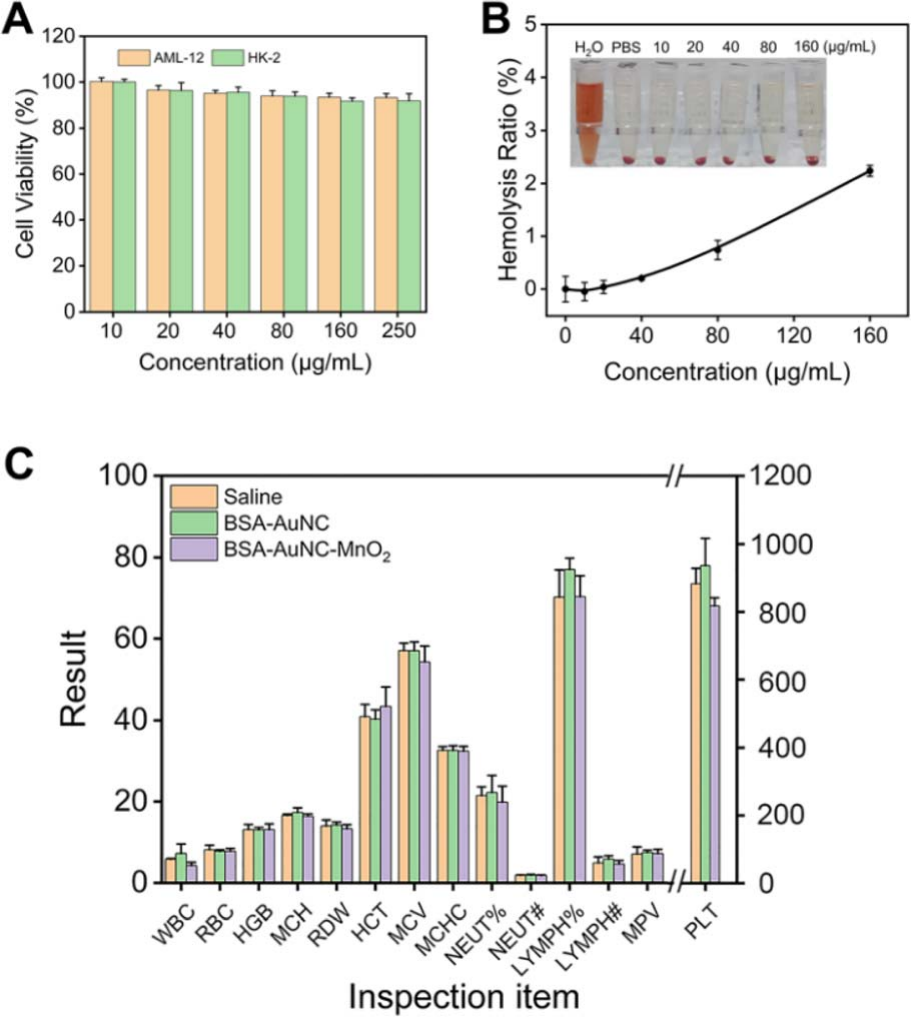
Biocompatibility and biosafety of BSA–AuNC–MnO_2_. (A) Cell viability of AML-12 and HK-2 cells after being incubated with different amounts of BSA–AuNC–MnO_2_ for 24 h. Data are means ± SD (*n* = 5). (B) Hemolysis ratio after being incubated with different concentrations of BSA–AuNC–MnO_2_ for 2 h. Data are means ± SD (*n* = 3). (C) Blood indexes of mice 48 h after intravenous injection of saline, BSA–AuNC and BSA–AuNC–MnO_2_. Data are means ± SD (*n* = 3).

After characterizing BSA–AuNC–MnO_2_, we next evaluated the biocompatibility and biosafety of BSA–AuNC–MnO_2_. Liver cell line AML-12 and kidney cell line HK-2 were chosen as the models for *in vitro* tests due to the non-specific accumulation of nanomaterials in the liver and kidney. BSA–AuNC–MnO_2_ induced negligible toxicity of both AML-12 and HK-2 cells even at the highest concentration (250 μg mL^−1^) as shown in Fig. 3A. Furthermore, BSA–AuNC–MnO_2_ caused minimal hemolysis (Fig. 3B), indicating the potential suitability of the system for systemic administration without significant adverse hematological effects. This finding underscores the importance of evaluating nanosystems for blood compatibility in the context of their safety *in vivo*. Further *in vivo* biocompatibility was supported by the comprehensive blood biochemistry panel (Fig. 3C). After administering BSA–AuNC–MnO_2_ nanoparticles through the tail vein, no significant differences were observed in a wide range of blood indexes, compared to the saline control group or reference values (Table S1†). Collectively, these results signify the exceptional biocompatibility of BSA–AuNC–MnO_2_ throughout the organism, thereby presenting a promising avenue for further therapeutic development and exploitation.

**Fig. 4:**
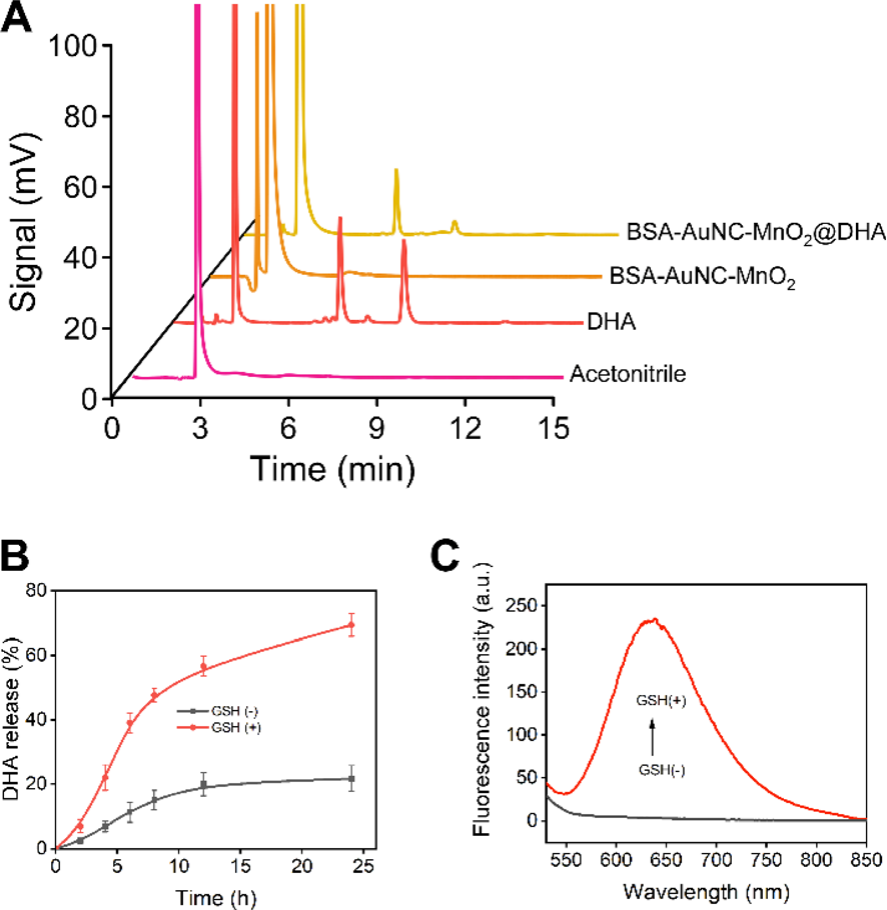
Characterization and responses to GSH of BSA–AuNC–MnO_2_@DHA. (A) HPLC analysis of acetonitrile, DHA, BSA–AuNC–MnO_2_, and BSA–AuNC–MnO_2_@DHA. (B) Release of DHA from BSA–AuNC–MnO_2_@DHA in the absence and presence of 2 mM GSH (*n* = 3). (C) Fluorescence emission spectra excited at 495 nm of BSA–AuNC–MnO_2_@DHA in the absence and presence of 2 mM GSH.

Subsequent to the thorough characterization, we proceeded with the encapsulation of DHA within the BSA–AuNC–MnO_2_ nanocomposite. This encapsulation process entailed utilizing a nanoprecipitation technique, wherein water was meticulously dripped into a solution of DHA and BSA–AuNC–MnO_2_ dissolved in acetone. The synthesized BSA–AuNC–MnO_2_@DHA nanoparticles displayed a uniform size of 79.9 ± 5.9 nm, verified by TEM (Fig. S11A†) and DLS (Fig. S11B†) analyses, indicating good homogeneity. The nanosystem, with a zeta potential of −28 mV (Fig. S11C†). BSA–AuNC–MnO_2_@DHA nanoparticles exhibit good biosafety both in normal (Fig. S11D†) and hypoxic conditions (Fig. S11E†). Furthermore, the nanosystem's tumor-targeting ability has been successfully observed in mouse imaging studies (Fig. S11F†), highlighting its potential as a promising platform for targeted drug delivery or imaging contrast agents in cancer therapy. The successful incorporation of DHA was substantiated by HPLC analysis, evinced by the distinctive peaks observed at 6.5 min and 9 min, respectively (Fig. 4A). Based on the calibration curve (Fig. S12 and Table S2†), the loading efficiency was calculated as 72.45% (Table S3†). Notably, the resultant BSA–AuNC–MnO_2_@DHA composite exhibited remarkable stability post-lyophilization, maintaining its integrity for a duration of at least 48 h (Table S4†). This stability is crucial for ensuring the longevity and effectiveness of the nanocomposite in practical applications. Furthermore, the release profile of DHA in an aqueous environment underscores the potential of this system for drug delivery applications. Specifically, in a PBS with a pH of 7.4, the release of DHA gradually approached a plateau after 12 h of incubation, with a cumulative release of less than 20% after 24 hours, indicating controlled and sustained drug release characteristics. The ideal manganese-based drug delivery system ought to maintain stability under normal physiological conditions (pH = 7.4) while exhibiting biodegradability within the tumor microenvironment. Consequently, two distinct dialysis media were formulated: the first was PBS buffer, which mimics the physiological environment of normal tissues (pH = 7.4), and the second was PBS buffer designed to simulate the tumor microenvironment (pH = 5.0, containing 2 mM GSH). A comprehensive investigation was conducted into the drug release characteristics of BSA–AuNC–MnO_2_@DHA nanocubes in these varying dialysis media (Fig. 4B). The findings revealed that under normal physiological conditions, the material released only 21.6% of DHA within 24 h. Conversely, within the tumor microenvironment, the release of DHA significantly escalated to 69.3%, suggesting that the drug release from BSA–AuNC–MnO_2_@DHA is environmentally responsive and that the tumor microenvironment serves as a catalyst for enhanced drug release. Furthermore, the elevated GSH levels not only triggered an intensified drug release but also instigated a resurgence of fluorescence emanating from BSA–AuNC (Fig. 4C). This dual-functional response, combining enhanced therapeutic efficacy with the capacity for fluorescence imaging, underscores the theranostic potential of the proposed system. By harnessing the unique chemical environment of tumor microenvironments, this nanoplatform adeptly integrates diagnostic and therapeutic modalities, offering a promising avenue for precision oncology. This comprehensive methodology guarantees the reproducibility, safety, and efficacy of the innovative BSA–AuNC–MnO_2_@DHA nanodelivery system for targeted cancer therapy. An exhaustive investigation into the incorporation of diverse concentrations of DHA into the BSA–AuNC–MnO_2_@DHA nanocomplex was conducted to meticulously evaluate the potential influence of the constituent synthetic materials, encompassing BSA, AA, HAuCl_4_, and MnCl_2_, on the quantitative determination of DHA content within the fabricated BSA–AuNC–MnO_2_@DHA nanostructure. The results indicate that the synthetic materials such as BSA, AA, HAuCl_4_, and MnCl_2_ do not exert a significant impact on the quantification of DHA within the BSA–AuNC–MnO_2_@DHA (Table S5†).

**Fig. 5:**
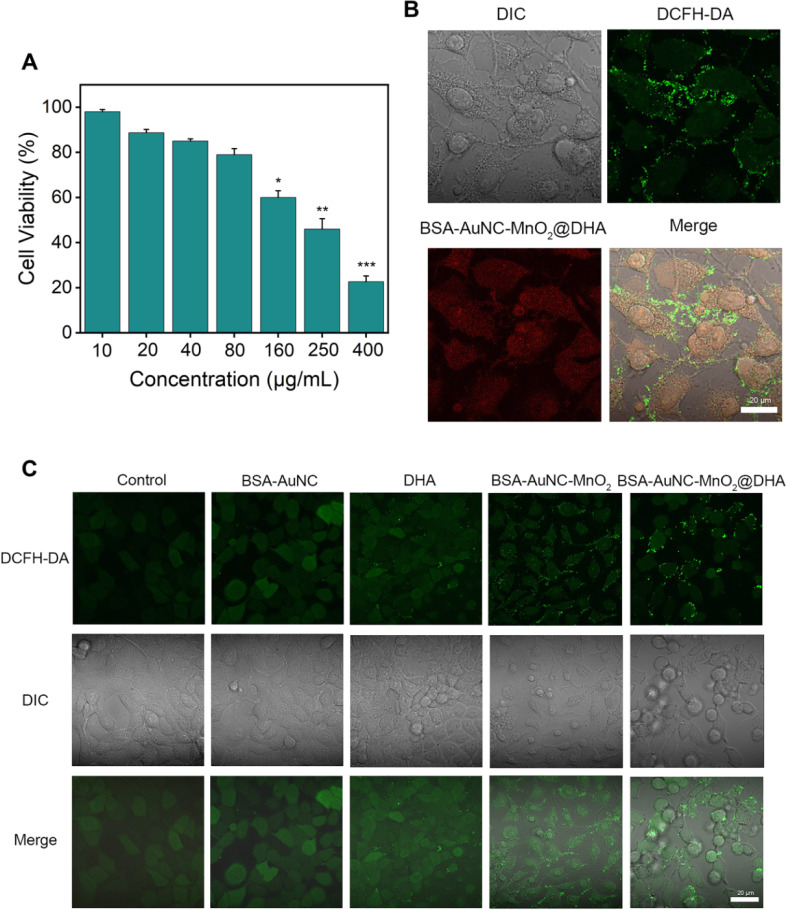
BSA–AuNC–MnO_2_@DHA induced cancer cell imaging and death *via* amplification of oxidative stress. (A) Cell viability of MCF-7 cells after being incubated with different concentrations of BSA–AuNC–MnO_2_@DHA for 24 h. Data are means ± SD (*n* = 5). (B) Fluorescence imaging of MCF-7 cells incubated with BSA–AuNC–MnO_2_@DHA and DCFH-DA as a ROS probe. Scale bar: 20 μm. (C) Intracellular generation of ROS using DCFH-DA as a probe in MCF-7 cells after different treatments. Scale bar: 20 μm.

The anticancer efficacy of BSA–AuNC–MnO_2_@DHA was rigorously evaluated in MCF-7 breast cancer cells, which served as a model system. Specifically, MCF-7 cells were incubated with varying concentrations of BSA–AuNC–MnO_2_@DHA for a period of 24 hours. Following this incubation, the cell viability was quantitatively assessed using the CCK-8 assay, a widely accepted methodology for determining cell proliferation and viability. The results presented reveal a clear trend of heightened cytotoxicity with increasing concentrations of BSA–AuNC–MnO_2_@DHA (Fig. 5A). Notably, at a concentration of 400 μg mL^−1^, a pronounced toxicity effect was observed, with only approximately 20% of the treated cells remaining viable. This finding underscores the potential of BSA–AuNC–MnO_2_@DHA as an effective anticancer agent, capable of inducing significant cell death in MCF-7 breast cancer cells at relatively high concentrations. Moreover, the dose-dependent nature of the observed toxicity highlights the significance of optimizing the concentration of BSA–AuNC–MnO_2_@DHA for therapeutic applications, with the aim of maximizing anticancer efficacy while minimizing potential adverse effects on non-cancerous cells. Therefore, the promising anticancer properties of BSA–AuNC–MnO_2_@DHA in MCF-7 breast cancer cells were demonstrated by the study. The cytotoxicity observed was found to be dose-dependent, with heightened toxicity occurring at higher concentrations, indicating the potential efficacy of this compound as an anticancer agent.

To comprehensively understand the mechanisms underlying cell death, we conducted a thorough investigation utilizing high-resolution confocal microscopy to accurately measure ROS levels. Our approach utilized the diacetyldichlorofluorescein (DCFH-DA) probe, which fluoresces upon reacting with ROS, providing a reliable indicator of ROS presence. The confocal imaging analysis revealed notable findings: the clear red fluorescence emanating from the BSA–AuNC–MnO_2_@DHA conjugates, due to the inherent fluorescence of the AuNCs, demonstrated successful cellular uptake (Fig. 5B). Furthermore, a distinct pattern of green fluorescence was observed in cells treated with BSA–AuNC–MnO_2_@DHA, indicative of an increased level of ROS generation compared to cells treated with DHA or BSA–AuNC–MnO_2_ alone (Fig. 5C). These findings strongly support the role of ROS in regulating cell death and highlight the effectiveness of our nanozyme platform in enhancing CDT efficacy through the encapsulation of DHA. The integration of DHA within the BSA–AuNC–MnO_2_ structure results in a significant increase in ROS production, positioning this approach as a promising candidate for further investigation in biomedical applications.

**Fig. 6:**
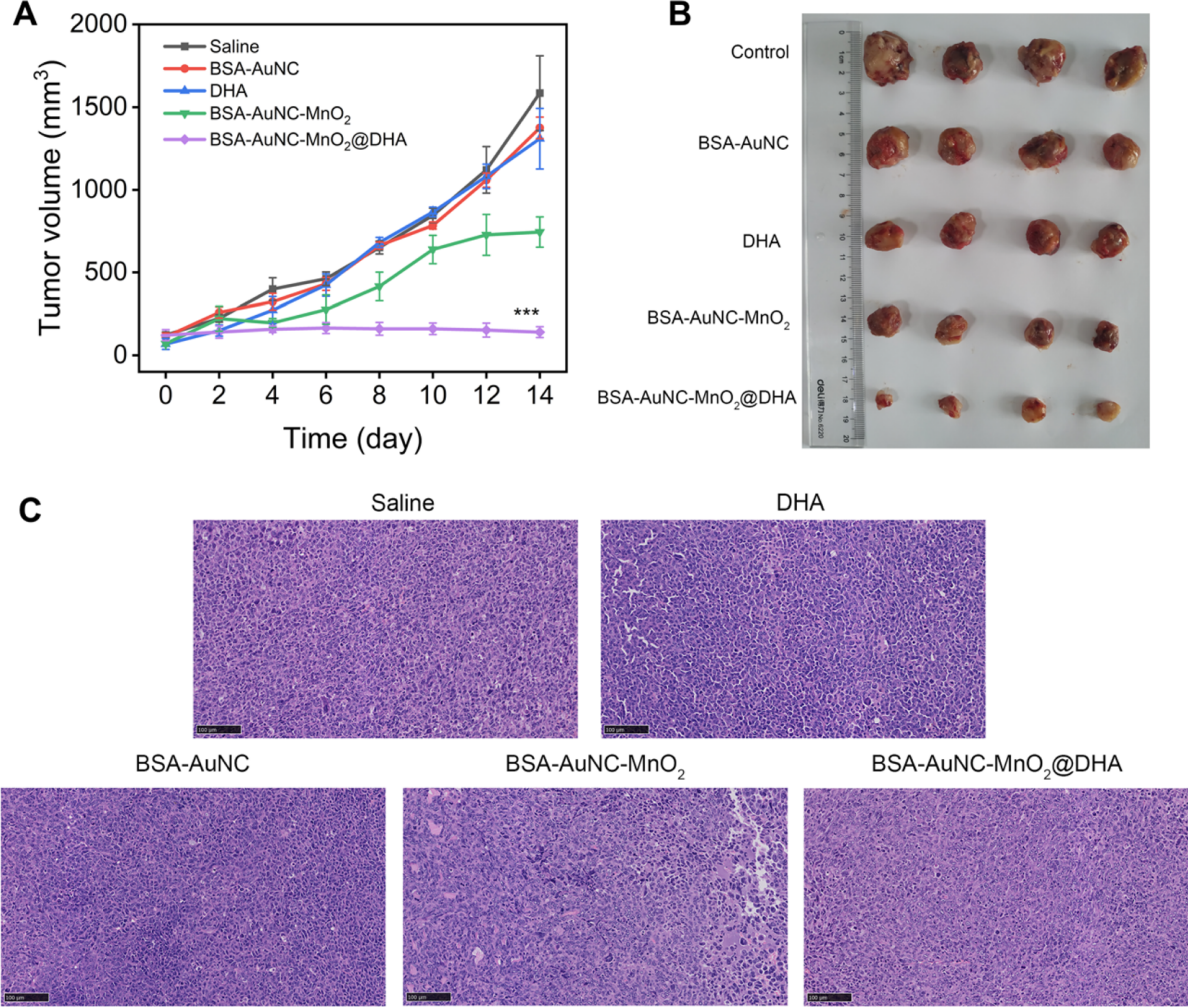
*In vivo* evaluation of anti-tumor efficacy. (A) Changes of tumor volume upon different treatments. Data are means ± SD (*n* = 6). Statistical analyses were performed using one-way ANOVA. ***P < 0.001 compared with other groups. (B) Photographs of tumors in the different medication administration. (C) Histological observation of the tumor tissues from different treatment groups stained with H&E. Scale bar: 100 μm.

Expanding upon the promising *in vitro* findings, we embarked on an *in vivo* evaluation of antitumor activity, utilizing mouse models to validate the therapeutic potential under more intricate biological milieus. LLC-tumor-bearing mice were meticulously assigned to five distinct treatment groups: (1) saline (serving as the control), (2) BSA–AuNC (to assess the contribution of the gold nanocluster component), (3) DHA (to evaluate the standalone effect of the drug), (4) BSA–AuNC–MnO_2_ (to examine the nanozyme's effect without DHA encapsulation), and (5) BSA–AuNC–MnO_2_@DHA (the complete therapeutic formulation). All treatments were administered *via* tail vein injection at a single dose of 0.9 mg kg^−1^. Tumor volume and body weight were meticulously monitored every two days over a 14 day period to comprehensively assess treatment efficacy and potential toxicity (Fig. S13†). BSA–AuNC–MnO_2_@DHA demonstrated a remarkable ability to inhibit tumor growth, with BSA–AuNC–MnO_2_ exhibiting a moderate yet significant ∼50% reduction (Fig. 6A). Conversely, BSA–AuNC and DHA alone failed to elicit any discernible therapeutic effect compared to the untreated group, underscoring the synergistic nature of the complete formulation. The profound tumor suppression observed with BSA–AuNC–MnO_2_@DHA can be attributed to its unique capacity for tumor-selective oxidative stress amplification. This conclusion is further bolstered by our findings, which revealed a starkly different response between cancerous and normal cells. The optical images of tumors from each treatment group provide visual evidence (Fig. 6B), directly corroborating the effectiveness of BSA–AuNC–MnO_2_@DHA in suppressing tumor growth. These results not only validate the potential of this innovative therapeutic strategy but also highlight the importance of rational drug design and targeted delivery in cancer treatment. The tumors were sectioned and stained with H&E for histological assessment (Fig. 6C). The observed cellular shrinkage and elevated nuclear density are indicative of apoptosis, demonstrating the efficacy of BSA–AuNC–MnO_2_@DHA in inducing cancer cell death. The uniform histological changes across sections suggest a robust antitumor response, emphasizing the potential of this nanocomposite as an effective anticancer agent.

**Fig. 7:**
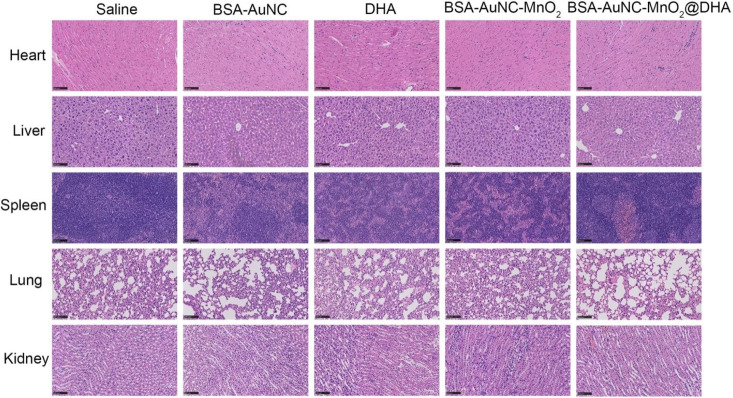
Histological observation of the organs from different treatment groups of mice stained with H&E. Scale bar: 100 μm.

The systemic effects of diverse treatment regimens on vital organs in mouse models were assessed (Fig. 7). Upon histological examination of hematoxylin and eosin (H&E)-stained tissue sections obtained from the treatment groups—comprising saline, BSA–AuNC, DHA, BSA–AuNC–MnO_2_, and the multimodal formulation BSA–AuNC–MnO_2_@DHA-notable signs of cellular damage or structural alterations were conspicuously absent. Notably, the cohort treated with BSA–AuNC–MnO_2_@DHA exhibited remarkable preservation of the normal tissue architecture in the heart, liver, spleen, lungs, and kidneys, indicative of a low level of systemic toxicity. This finding is particularly noteworthy in light of the previously demonstrated anti-tumor efficacy of BSA–AuNC–MnO_2_@DHA, as evidenced by Fig. 6. The absence of significant histopathological alterations serves as an encouraging indication that the therapeutic benefits of BSA–AuNC–MnO_2_@DHA can be realized without compromising the health of crucial organs. This promising safety profile underscores the potential of BSA–AuNC–MnO_2_@DHA as a viable candidate for cancer treatment. To further validate its safety and efficacy, extended toxicity studies and an in-depth exploration of its antitumor mechanisms are warranted. Such endeavors will not only enrich our understanding of BSA–AuNC–MnO_2_@DHA but also facilitate the development of safer and more efficacious cancer therapies.

## Conclusions

Drawing upon the capability of bovine serum albumin (BSA) to form cubic structures under thermal conditions, we have devised a template-guided strategy, inspired by natural biomineralization processes, for metal ion immobilization and metal nanocube synthesis. By precisely adjusting the material composition, reductant concentration, pH, temperature, and reaction time, we have developed a streamlined and adaptable method for producing both monometallic gold nanocubes and bimetallic gold-manganese nanocubes. This approach offers improvements over traditional synthesis methods in terms of simplicity and efficiency. Using this synthesis methodology, we have successfully created a multifaceted drug carrier, BSA–AuNC–MnO_2_, which displays cubic morphology, consistent particle size distribution, good stability, and acceptable biocompatibility. Notably, this carrier exhibits responsiveness to the tumor microenvironment, facilitating the enhancement of chemotherapy kinetics and enzymatic therapeutic strategies. Therefore, it represents a potentially valuable tool for therapeutic applications. To overcome the limitations of DHA in terms of solubility and delivery efficiency, we have encapsulated DHA within the BSA–AuNC–MnO_2_ carrier, resulting in the BSA–AuNC–MnO_2_@DHA metallic nanodrug delivery system. This formulation improves DHA's solubility and delivery, enabling a cooperative interaction between the drug and the nanocarrier that promotes oxidative stress. The performance of this system has been thoroughly evaluated and demonstrated effectiveness at both cellular and *in vivo* levels.

## Ethical statement

The animal experiments were performed according to an approved agreement by the Institutional Animal Ethics Committee of China Pharmaceutical University (SYXK2021-0010).

## Data availability

The authors confirm that the data supporting the findings of this study are available within the article and/or its ESI.†

## Author contributions

W. Z., L. L. and J. T.: conceptualization, data curation, formal analysis, investigation, conceptualization, supervision. A. T. W. and X. W.: data curation, formal analysis, methodology, writing-original draft. S. D.: methodology, formal analysis.

## Conflicts of interest

A. T. W. was an intern at Shanghai Xianguang Biotechnology Co., Ltd. S. D. and L. L. are current or past employees of Shanghai Xianguang Biotechnology Co., Ltd.

## Supplementary Material

RA-014-D4RA03164D-s001
