# Supplementary material for: A gold cluster fused manganese dioxide nanocube loaded with dihydroartemisinin for effective cancer treatment via amplified oxidative stress
Source: RSC Adv. 2024 Sep 2;14(38):27703–11. doi: 10.1039/d4ra03164d (PMC11367086; doi:10.1039/d4ra03164d)
Supplement: RA-014-D4RA03164D-s001 [file RA-014-D4RA03164D-s001.pdf]

Supplementary Information (SI) for RSC Advances.
This journal is © The Royal Society of Chemistry 2024

Supporting Information

A Gold Cluster Fused Manganese Dioxide Nanocube Loaded with Dihydroartemisinin for Effective Cancer Treatment via Amplified Oxidative Stress

Alan Tianyi Wang†[a,b], Xin Wen†[a], Shangyi Duan[b], Jiangwei Tian*[a], Liang Liu*[b], and Wangning Zhang*[a]

a. State Key Laboratory of Natural Medicines, Jiangsu Key Laboratory of TCM Evaluation and Translational Research, School of Traditional Chinese Pharmacy, China Pharmaceutical University, 639 Longmian Avenue, Nanjing, 211198, China. Email: jwtian@cpu.edu.cn; 18435148209@163.com

b. Shanghai Xianguang Biotechnology Co., Ltd, 318 Jungong Road, Shanghai, 200090, China. Email: stephen@xgbiotech.com

†These authors contributed equally to this work.

Supplementary Experiments

Reagents

Ascorbic acid (AA) was purchased from Sinopharm Reagent Co., Ltd. (Shanghai, China). $MnCl_2$ and Ethylene Diamine Tetraacetic Acid (EDTA) were acquired from Macklin Biochemical Co., Ltd. (Shanghai, China). DHA, $HAuCl_4 \bullet 3H_2O$, 3,3',5,5'-Tetramethyl-benzidine (TMB), methylene blue (MB) and glucose were obtained from Aladdin Co. Ltd. (Shanghai, China). Bovine albumin (BSA) was provided by Biofroxx (Germany). Fetal bovine serum (FBS) was obtained from Procell Life Science & Technology Co., Ltd. (Wuhan, China). Dulbecco's modified eagle medium (DMEM), phosphate buffered saline (PBS) and 0.25% Trypsin were supplied from KeyGen Biotech. Co. Ltd. (Nanjing, China). Triton X-100 was purchased from Sigma-Aldrich (St. Louis, MO, USA). Cell Counting Kit-8 (CCK-8) was obtained from Biosharp. ROS Detection Kit was purchased from Beyotime Biotechnology Co., Ltd. (Shanghai,China). Ultrapure water was prepared using a Millipore Simplicity System (Millipore, Bedford, USA)

Apparatus

The morphology and size of MOFs were observed by JEM-2100 transmission electron microscopy (TEM, Japan). A FT-IR spectrometer (PerkinElmer, USA) was applied to obtain the Fourier transform infrared (FT-IR) spectra. The X-ray photoelectron spectroscopy (XPS) data was obtained from an X-ray photoelectron spectrometer (K-Alpha, Thermo Scientific, America). Powder X-ray diffraction (PXRD) spectra were determined by an X'Pert PRO MPD X-ray diffractometer (Nalytical, Netherlands). Fluorescence spectrum and ultraviolet spectrum were measured by a FL-4600 fluorescence spectrometer (Hitachi, Japan) and an UV-2600 ultraviolet spectrometer (Shimadzu, Japan), respectively. All the NMR spectroscopy data was obtained from Nuclear magnetic resonance spectrometer (Bruker, Switzerland). A hand-held 254 nm UV lamp (ZF-7A, Shanghai, China) was designed as a part of portable and visual device,

and visual ALP assay was performed by the visual device. All experiments were performed at room temperature.

Method section

Synthesis of BSA-AuNC. The synthesis of gold nanoclusters AuNCs was performed based on a previously reported method. Gold nanoclusters AuNCs were prepared by the reduction of $HAuCl_4$ using BSA and ascorbic acid (AA). BSA served as the reducing and simultaneously stabilizing agent and the whole reduction was performed in an alkaline environment (pH=12). In a typical procedure, a mixture of BSA (100 mg) and $HAuCl_4$ (100 μL, 500 mM) in Milli-Q water (10 mL) was vigorously stirred. Then, AA (100 μL, 2 mM) was dropwise added under stirring. Afterward, NaOH (1 M) was added to adjust the solution pH to 12, and the reaction was allowed to proceed under stirring for 2 h at the temperature of 100 °C. After cooling to room temperature, the products were dialyzed using a 14 kDa dialysis bag against Milli-Q water for 24 hours and then stored at 4 °C (fridge) for later use.

Synthesis of BSA-AuNC-MnO_2. The BSA-AuNCs were prepared as templates for synthesizing the Au&Mn nanocube (BSA-AuNC-MnO_2). $MnCl_2$ and $HAuCl_4$ were used as sources of Mn and Au. The effects of Mn^{2+} on the synthesis of BSA-AuNC-MnO_2 were determined at concentrations ranging from 0 to 7 mM. The best one (3 mM) was synthesized by using BSA (100 mg), $MnCl_2$ (150 μL, 200 mM), and $HAuCl_4$ (100 μL, 500 mM) in Milli-Q water (10 mL). Besides, all the other procedures were similar to those described above of BSA-AuNC. The final solution was stored at 4 °C after extensive dialysis against Milli-Q water.

Synthesis of BSA-AuNC-MnO_2@DHA. BSA-AuNC-MnO_2@DHA was prepared using the modified nanoprecipitation method. In brief, BSA-AuNC-MnO_2 was first lyophilized. A mixture of DHA (5 mg) and freeze-dried BSA-AuNC-MnO_2 (10 mg) in acetone (5 mL) was stirred at 37 °C for several hours until most of the solvent was evaporated. Subsequently, Milli-Q water was slowly added dropwise under constant

stirring at 800 rpm. The solution was stirred under the same conditions until all acetone evaporated to obtain BSA-AuNC-MnO_2@DHA.

Characterization. The BSA-AuNC and BSA-AuNC-MnO_2 were characterized to determine morphology, elemental composition, particle size, surface charge, and fluorescence. The samples were dropped onto a carbon-coated copper grid for TEM measurement. The freeze-dried samples were degassed at 80 °C under vacuum for X-ray photoelectron spectroscopy (XPS). The loading capacity of DHA was determined using the HPLC method with the detection wavelength of 216 nm. All determinations were performed at least three times.

Loading efficiency (LE) and *in vitro* release capacity of BSA-AuNC-MnO_2@DHA. The LE of DHA was evaluated by the total amount of DHA in the nanocube. 10% Triton X-100 was added to the sample solution with sonicating for 30 min. Next, 0.5 mL of BSA-AuNC-MnO_2@DHA dispersion was ultrafiltered through an ultrafiltration tube at 8000 rpm for 15 min. The collected filtrate was analyzed by HPLC to calculate the drug loading efficiency. The LE of content was calculated as LE (%) = $W_1/(W_1+W_2)\times 100\%$, where W_1 and W_2 were the weights of loaded content and unloaded content, respectively.

The release capacity determination of BSA-AuNC-MnO_2@DHA nanoparticles was performed by a dialysis method in vitro. One milliliter of BSA-AuNC-MnO_2@DHA suspension (1 mg/mL) was dialyzed against 15 mL pH 7.4 PBS solution using a 14 kDa dialysis bag at 37 °C. At various time points, aliquots of 3.0 mL were withdrawn and immediately replaced with the same volume of fresh release media. The DHA content of the withdrawn samples was determined by HPLC.

***In vitro* stability and enzyme-like catalysis.** The physiological environment stability of samples was performed by using laser granulometry. Prepared solutions were incubated with saline, PBS, DMEM, and 10% Fetal Bovine Serum (FBS) at room temperature, respectively. The hydrodynamic diameters of samples in different physiological environments were tested within one week to evaluate the physiological environment stability of BSA-AuNC and BSA-AuNC-MnO_2 nanoparticles. The changes in the fluorescence spectra of BSA-AuNC and in UV absorption of BSA-AuNC-MnO_2

were monitored between samples freshly prepared and those stored in a refrigerator at 4 °C for 30 days.

The generation of •OH was monitored by the decay of methylene blue (MB) in the characteristic absorption peak. The experimental solution contains 1 mL of $NaHCO_3$ (250 mM), 10 μL of H_2O_2 (1M), 10 μL of MB (1 mg/mL), and 200 μL of BSA-AuNC-MnO_2 with variable GSH solution (0 μL, 5 μL, 10 μL, 100 μL). Pure MB aqueous solution (10 μg/mL) was used as the control experiment. The solutions were incubated at 37 °C for 0.5 h prior to the reading.

Cell culture. The mouse normal liver AML-12 cell line, human renal tubular epithelial HK-2 cell line, mouse lung cancer LLC cell line and human breast cancer MCF-7 cell line were obtained from KeyGen Biotech Co. Ltd. (Nanjing, China). All cell lines were cultured in DMEM containing 10% FBS, 100 $\mu g•mL^{-1}$ streptomycin and 100 $U•mL^{-1}$ penicillin at 37 °C in a humidified incubator containing 5% CO_2 and 95% air. The medium was replenished every other day and the cells were sub-cultured after reaching confluence.

Animals and tumor model. BALB/c male and C57BL/6 male mice (18-20 g) were purchased from Jinan Pengyue Laboratory Animal Breeding Co., Ltd. (Animal licence No. SCXK 20190003). The animal experiments were performed according to an approved agreement by the Institutional Animal Ethics Committee of China Pharmaceutical University (SYXK2021-0010). Additionally, all laboratory animal procedures were conducted in adherence to the guidelines outlined in the Guide for the Care and Use of Laboratory Animals issued by the Ministry of Science and Technology of China. LLC tumor model was established by subcutaneous injection of LLC cells (1×10^6) into the armpit of the C57BL/6 male mice with further culture to become a solid tumor for therapeutic experiments.

Hemolysis and blood routine. 2 mL of BALB/c mice blood (orbital venous plexus blood collection) was prepared into a 2% RBC suspension. 1 mL PBS solution containing serial concentrations of BSA-AuNC or BSA-AuNC-MnO_2 (experimental groups), 1 mL of Milli-Q water (positive control), and 1 mL of pH=7.4 PBS buffer

(negative control) were incubated with 0.4 mL of 2% RBC suspension at 37 °C for 2 h. Next, the resulting suspensions were centrifuged at 3600 rpm for 5 min. The absorbance of the supernatant was measured at 540 nm to reflect the amount of hemoglobin released.

For further evaluating the biocompatibility of nanoparticles *in vivo*, BSA-AuNC or BSA-AuNC-MnO_2 nanoparticles (10 mg/kg) and normal saline were injected into the corresponding groups of mice through tail veins, respectively. The blood was collected from the mice at 48 h for evaluation of toxicity through blood tests.

Cytotoxicity assay. To investigate the cytotoxicity of BSA-AuNC, BSA-AuNC-MnO_2 and BSA-AuNC-MnO_2@DHA, CCK-8 assays were performed on AML-12, HK-2 and MCF-7 cells. Briefly, AML-12, HK-2 and MCF-7 cells were separately seeded at a density of 3×10^3 cells per well in 96-well plates in 100 μL of complete medium and incubated at 37 °C for 24 h. After rinsing with PBS, AML-12 and HK-2 cells were incubated with 100 μL culture media containing serial concentrations of BSA-AuNC or BSA-AuNC-MnO_2 for 24 h, respectively. MCF-7 cells were incubated with 100 μL culture media containing serial concentrations of BSA-AuNC-MnO_2@DHA for 24 h. The blank control was incubated with the same volume of DMEM. Subsequently, 20 μL of CCK-8 was added to each well and incubated for 3 h. Next, absorbance at 450 nm was measured with a microplate reader to calculate the cell viability.

Intracellular ROS generation. MCF-7 cells (about 5×10^5 cells per plate) were seeded into plates 24 h prior to the experiment. BSA-AuNC, DHA, BSA-AuNC-MnO_2 and BSA-AuNC-MnO_2@DHA were incubated cells for 24 h followed by adding 2 mL of DCFH-DA (10 μM in DMEM) was added and cultured for another 30 min. Cells were imaged using confocal microscopy at 488 nm for excitation and at 510-560 nm for emission.

In Vivo **Study of Antitumor Efficacy.** For investigating the antitumor effect *in vivo*, LLC tumor-bearing mice were subjected to five different treatments through tail vein injections in the following groups: saline control; BSA-AuNC; DHA; BSA-AuNC-MnO_2 and BSA-AuNC-MnO_2@DHA. A single dose of each formulation was administered into the tail vein at 0.9 mg•kg^{-1}, once every two days for 14 days. During the experiment, the

tumor volume and body weight of each mouse were measured and recorded every two days. After 14 days of treatment, the mice were euthanized. Major organs including heart, liver, spleen, lung, kidney, and tumor were removed, followed by washing the surface of each tissue with physiological saline several times, fixed in formalin solution (4%), cut into 5 μm sections, and embedded in paraffin after H&E staining, which were observed through fluorescence microscopy.

Statistical Analysis. The obtained data were all statistically analyzed using OriginPro 2021.Experimental data are presented as mean ± standard error (mean ± SEM). Student's test was used to test the data statistics between two groups, and Dunnett's test was used after one-way ANOVA when there were three or more groups. $P<0.05$ was considered to be statistically significant.

Supplementary Figures

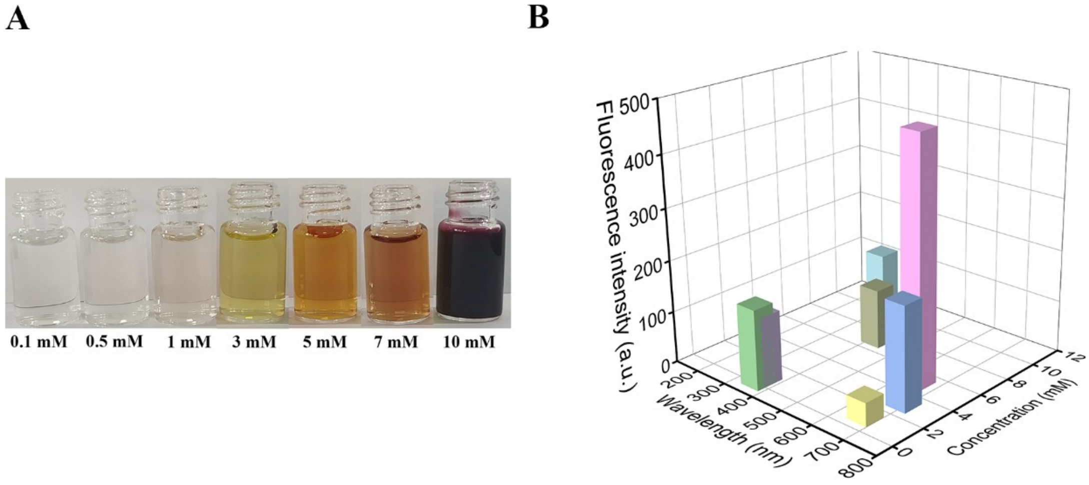

Figure S1. Effect of $HAuCl_4$ concentration on synthesis of BSA-AuNC in the presence of 10 mg/mL. (A) Photographs of BSA-AuNC synthesized at different concentrations ratios of $HAuCl_4$. (B) Fluorescence intensity of BSA-AuNC synthesized at different concentrations of $HAuCl_4$.

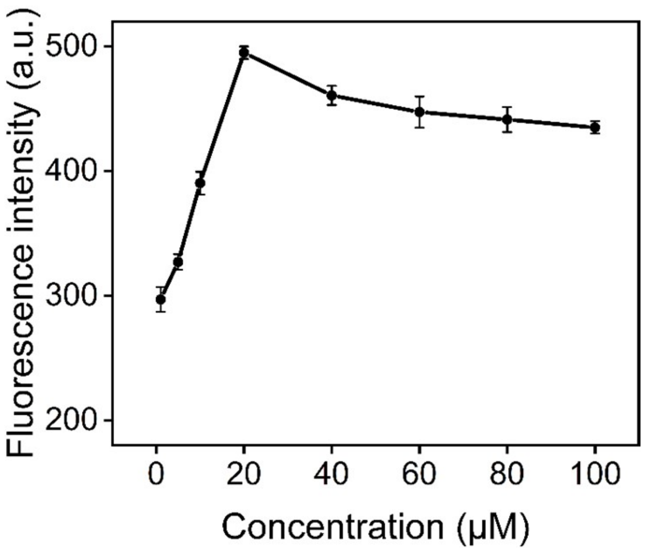

Figure S2. Fluorescence intensity of BSA-AuNC synthesized at different concentrations of AA ($\lambda_{ex/em}$=495/650 nm).

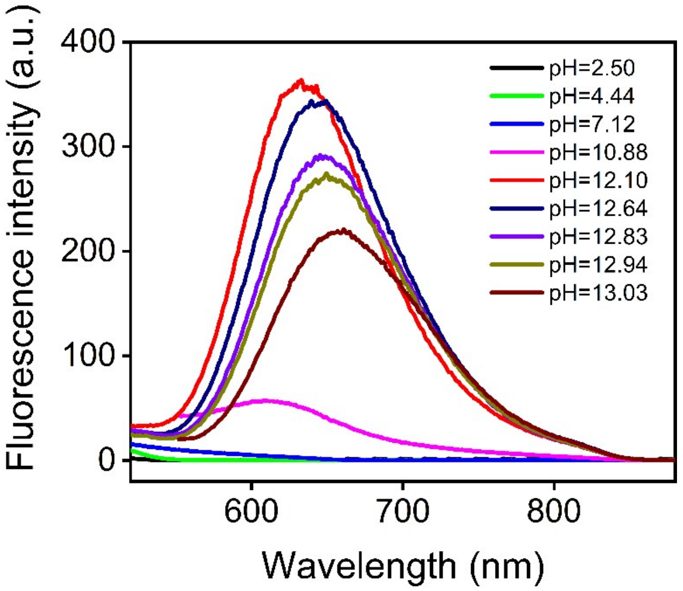

Figure S3. Fluorescence emission spectra of BSA-AuNC synthesized at different pH values (λ_{ex}=495 nm).

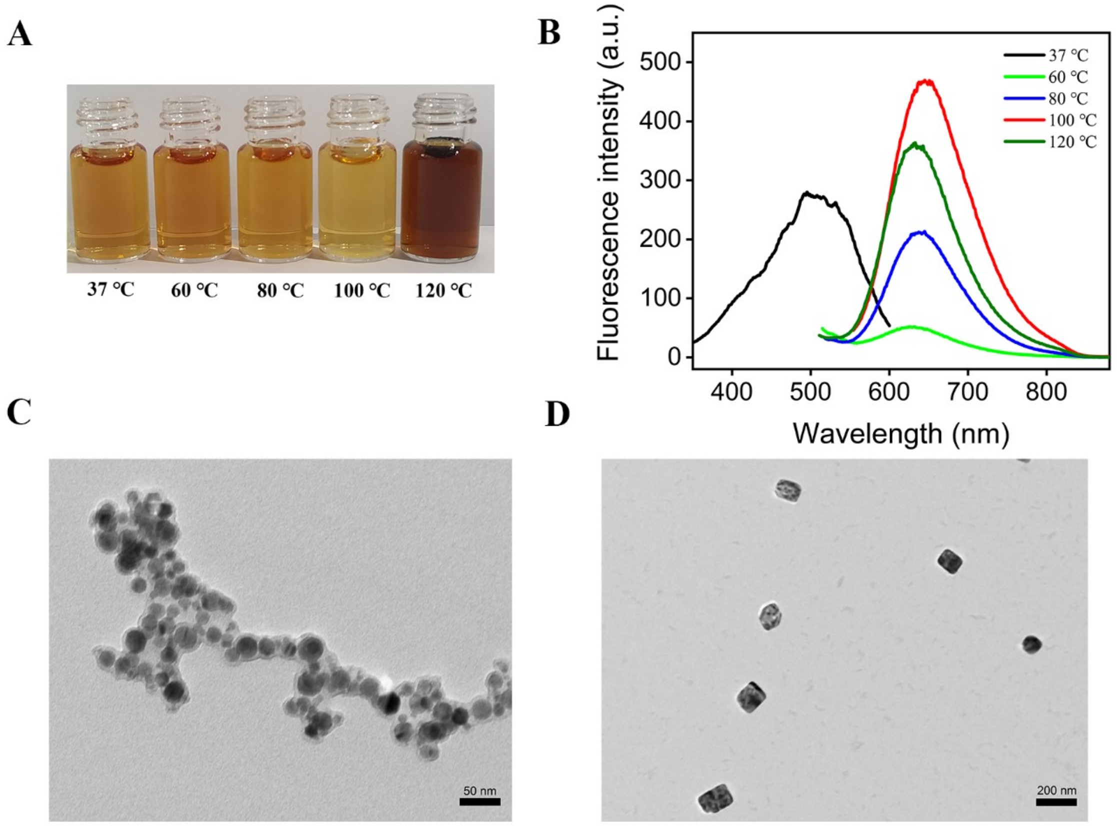

Figure S4. Effect of reaction temperature on synthesis of BSA-AuNC. (A) Photographs of BSA-AuNC synthesized at different temperatures. (B) Fluorescence excitation and emission spectra of BSA-AuNC synthesized at different temperatures. (C) TEM image of BSA-AuNC synthesized at 37 °C. Scale bar: 50 nm. (D) TEM image of BSA-AuNC synthesized at 100 °C. Scale bar: 200 nm.

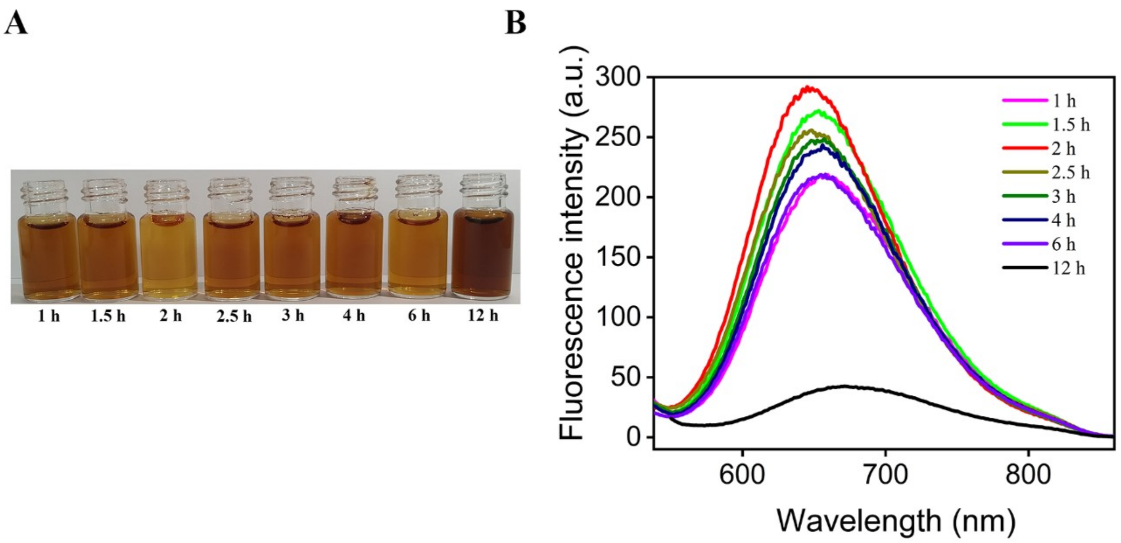

Figure S5. Effect of reaction time on the synthesis of BSA-AuNC. (A) Photographs of BSA-AuNC synthesized at different reaction times. (B) Fluorescence excitation spectra of BSA-AuNC synthesized at different reaction times (λ_{ex}=495 nm).

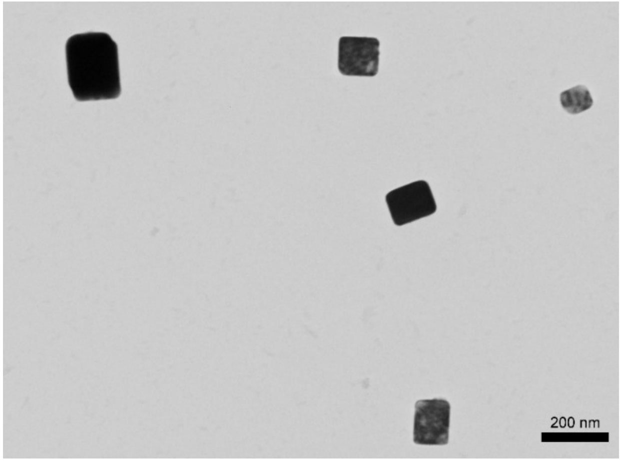

Figure S6. TEM image of BSA-AuNC. Scale bar: 200 nm.

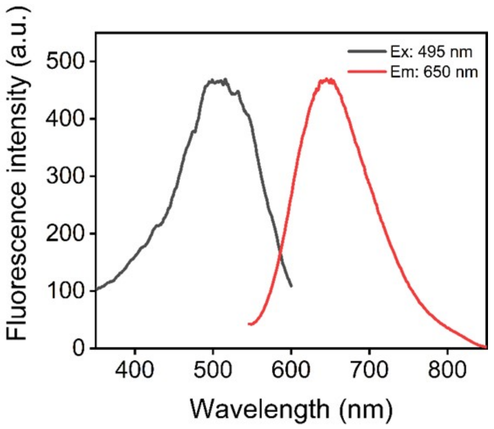

Figure S7. Fluorescence excitation and emission spectra of BSA-AuNC.

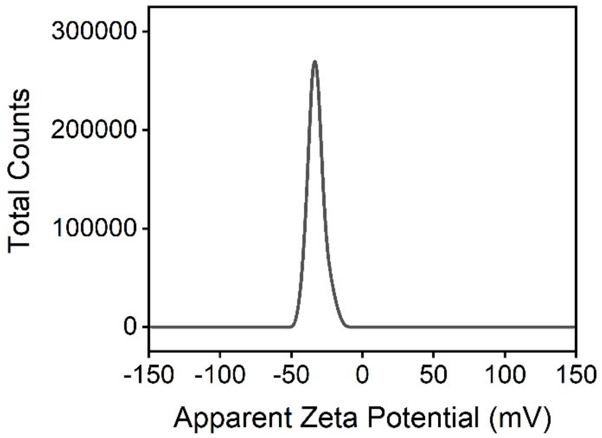

Figure S8. Zeta potential of BSA-AuNC-MnO_2.

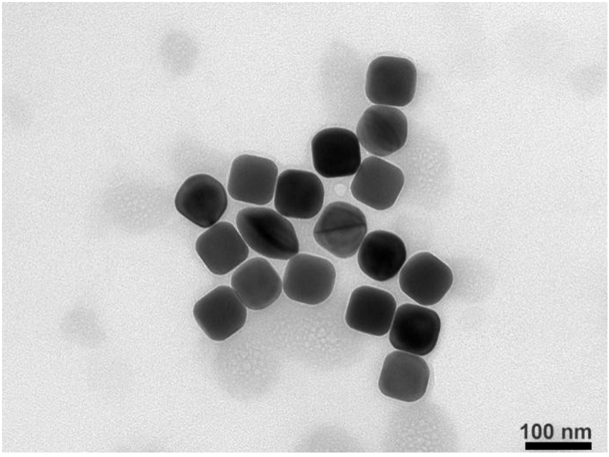

Figure S9. TEM image of BSA-AuNC-MnO_2 (scale bar= 100 nm).

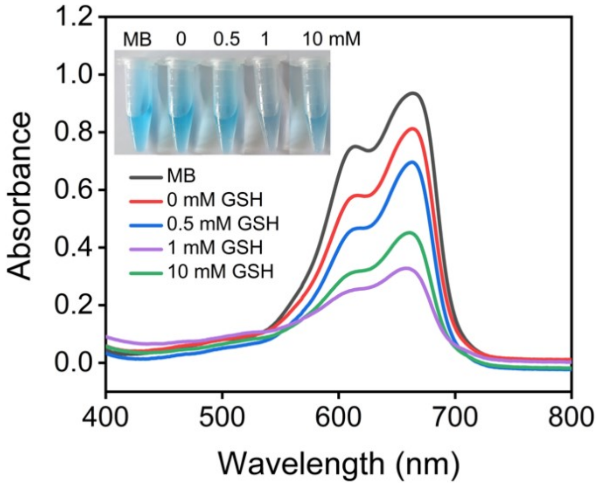

Figure S10. UV-vis absorption spectra of methylene blue (MB) under different reaction conditions (black line: MB; red line: MB + BSA-AuNC-MnO_2 + H_2O_2 + 0 mM GSH; blue line: MB + BSA-AuNC-MnO_2 + H_2O_2 + 0.5 mM GSH; purple line: MB + BSA-AuNC-MnO_2 + H_2O_2 + 1 mM GSH; green line: MB + BSA-AuNC-MnO_2 + H_2O_2 + 10 mM GSH).

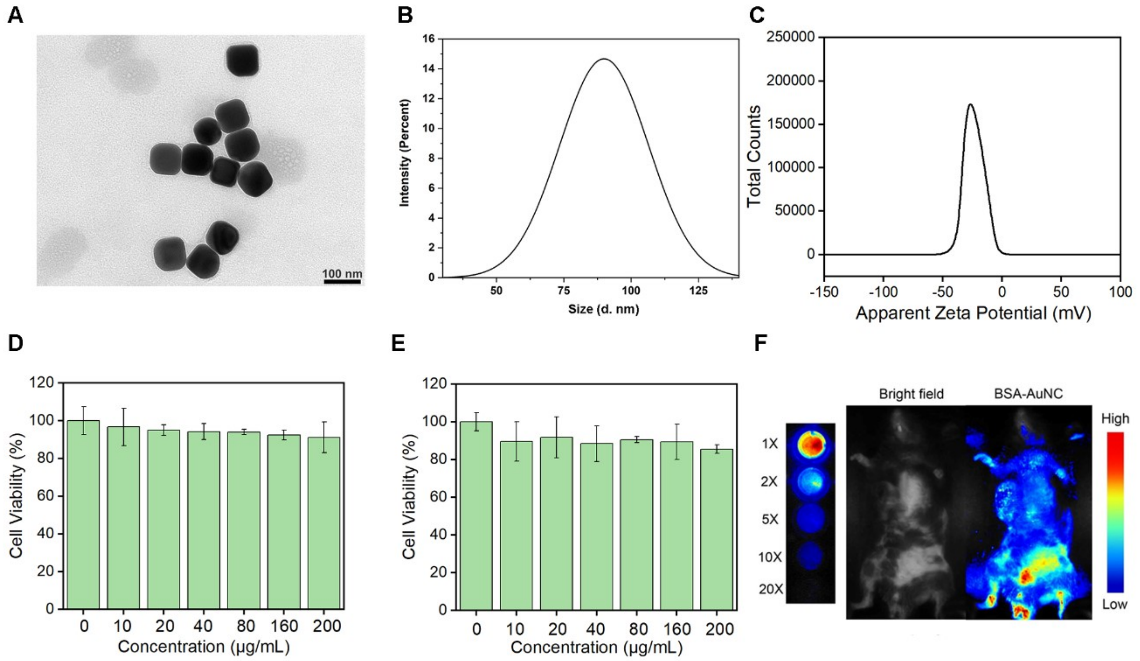

Figure S11. The physicochemical and biological characterizations of BSA-AuNC-MnO_2@DHA. (A) TEM, (B) DLS, (C) Zeta potential image of BSA-AuNC-MnO_2@DHA (scale bar = 100 nm). Cell viability of HK-2 cells after being incubated with different amounts of BSA-AuNC-MnO_2@DHA for 12 h under (D) normal and (E) hypoxic conditions. Data are means ± SD (n = 5). (F) In vivo imaging of BSA-AuNC-MnO_2@DHA in mice (λex = 523 nm).

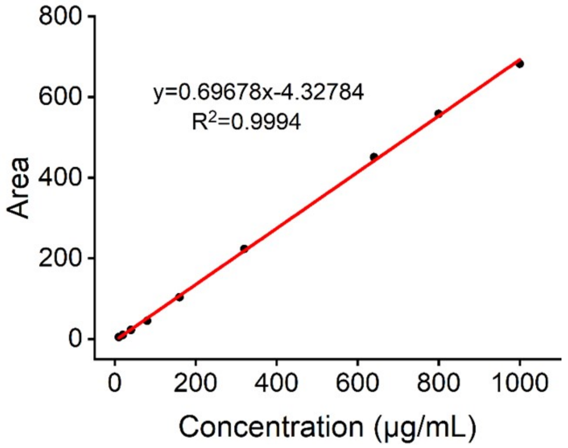

Figure S12. The standard curve of DHA is determined by HPLC. The obtained standard curve is y=0.069678x-4.32784 (y: integral area of DHA; x: concentration of DHA, R^2 =0.9994).

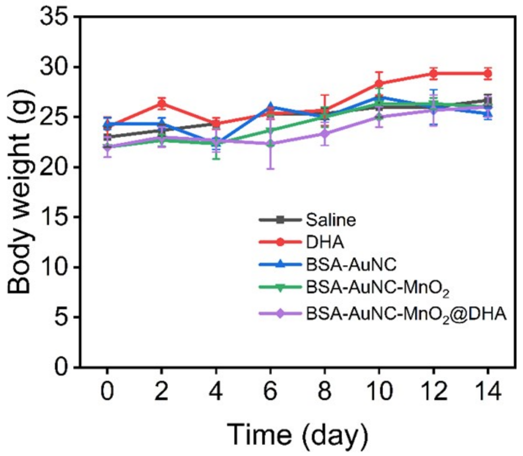

Figure S13. Body weight changes of LLC tumor-bearing mice during different treatments. Data are means ± SD (n = 6).

Supplementary Tables

Table S1 Blood routine inspection items and reference values

Test items	Abbreviation	Content unit	Reference interval/value
Leukocyte	WBC	10^9/L	3.2-12.7
Red blood cells	RBC	10^{12}L	7.0-11.0
Hemoglobin	HGB	g/dL	11.8-14.9
Hemoglobin amount	MCH	pg	13.8-18.4
Red blood cell distribution width	RDW	%	11.7-15.1
Hematocrit	HCT	%	36.7-46.8
Mean red blood cell volume	MCV	fL	42.2-59.2
Mean hemoglobin concentration	MCHC	g/dL	31.0-34.7
Neutrophil ratio	NEUT%	%	6.8-31.1
Neutrophil absolute value	NEUT#	10^9/L	0.5-2.0
Lymphocyte ratio	LYMPH%	%	60.2-95.0
Absolute value of lymphocytes	LYMPH#	10^9/L	3.8-8.9
Mean platelet volume	MPV	fL	5.0-8.0
Platelets	PLT	10^9/L	766-1657

Table S2 Relationship between peak area and content of DHA

Concentration (μg/mL)	Area
1000.00	682.21
800.00	558.01
640.00	450.89
320.00	223.17
160.00	103.73
80.00	45.11
40.00	22.34
20.00	10.05
10.00	4.66

Table S3 Encapsulation efficiency of DHA loaded in BSA-AuNC-MnO_2

No.	C_{DHA} (μg/mL)	EE (%)	Average (%)	RSD (%)
1	721.24	72.10		
2	738.23	73.82	72.45	1.69
3	714.74	71.44		

Table S4 Stability test results

Sample	0 h	8 h	16 h	24 h	36 h	48 h	RSD (%)
BSA-AuNC-MnO_2@DHA	702.51	704.17	712.62	698.57	684.90	701.49	1.30

Table S5 Sample recovery test results.

Sample	Area	Concentration (μg/mL)	Recovery (%)	RSD (%)
BSA-AuNC-MnO_2@DHA	485.83	703.46	/	/
BSA-AuNC-MnO_2@DHA+50 μg DHA	519.38	751.61	96.30	2.36
BSA-AuNC-MnO_2@DHA+100 μg DHA	555.93	803.93	100.47	2.25
BSA-AuNC-MnO_2@DHA+200 μg DHA	624.82	902.94	99.74	2.66
